# Hospital Meetings, &c.

**Published:** 1899-06-17

**Authors:** 


### HOSPITAL MEETINGS, &C.

### CITY OF LONDON HOSPITAL FOR DISEASES OF THE CHEST.

The annual festival dinner in connection with the City of London Hospital for Diseases of the Chest, Victoria Park, E., was held at the Criterion Restaurant, W., on Wednesday, the 7th inst., under the presidency of the Hon. Walter Rothschild, M.P. Among those present were: Dr. Heron, Ven. Archdeacon Sinclair, D.D., Sir Edward Sassoon, Bart., M.P., Mr. Joseph Benson, L.C.C., Mr. H. J. Allcroft, Mr. Herbert Robertson, M.P., Mr. Philip Savill, Mr. Horace G. Bowen, Dr. C. A. Coleman, Mr. Frank Hyland, Dr. Clifford Beale, Mr. Worthington Evans, Mr. E. E. Cooper, Rev. J. E. Watts Ditchfield (chaplain), Mr. H. T. Dudley Ryder (secretary), Rev. Father Denny, Mr. F. Garland, Dr. Walsham, Mr. S. S. Baiss, Dr. Chaplin, Mr. H. Tuck, Dr. Colbeck, Dr. Emanuel, Dr. Glover Lyon, Dr. Rennie, Dr. Williamson, Dr. Perkins, Dr. Blaker, Mr. J. D. Farrell, Dr. Harrington Sainsbury, Mr. John Norbury, jun., Mr. H. Moy, Mr. Julius Lewisohn, Mr. Samuel Moses, Mr. A. Fisher, and representatives of the Good Intent Aid Society, Bethnal Green Society, North Bow Society, Bethnal Green Working Men's Society, Mutual Friendly Aid Society, St. James's Philanthropic Society, Lion Hospital Society, V.A.O. Druids, Excelsior Philanthropic Society, Mile End New Town Society, Haggerston Hospital Society, and the Albert and Victoria Society.

After the customary loyal toasts,

Mr. Philip Savill submitted "The Houses of Parliament." This was suitably acknowledged by Mr. Herbert Robertson, M.P., who alluded to the fact that only a day or so previously the assistance of a great metropolitan hospital had been sought on behalf of one of the members of the House of Commons.

The Chairman, in proposing "Prosperity to the City of London Hospital for Diseases of the Chest," said he thought everyone would acknowledge that a hospital was one of the most important, if not the most important, forms of charity. It was one of those institutions that must command their sympathies, energies, and purses to further its objects, because those objects were first of all, and almost entirely, the relief of suffering and misery; and another aspect from which the hospitals should command their hearty support was that they were great centres of medicine, enabling doctors and surgeons by their observation to find out new means of curing disease and thus alleviate suffering. To turn to this special institution, as was probably known to most present, it began its life of usefulness more than fifty years ago in a small dispensary near Liverpool Street, which had long since been swept away. The foundation-stone of the present building was laid by the lamented Prince Consort. As the population around it increased the building was completed, and he did not think any metropolis could wish for a finer institution. Nearly 32,000 in-patients and over 600,000 out-patients had been relieved since the beginning of the hospital. (Applause.) There were under treatment on January 1st, 1898, over 1,000 out-patients, and the attendances of out-patients during the past year amounted to between 63,000 and 64,000. In the East-end labourers, artisans, and workers of all kinds were exposed from morning till night to all conditions of weather and life, and that fact alone showed the need of such an institution, and should inspire those present to open wide their purse strings and aid it with all the power they possibly could. One great point in favour of the institution was that patients were accepted irrespective of nationality or creed. As recently as 1897 out of the 160 beds in the hospital the funds at the disposal of the management were only sufficient to open 16, the hospital being in straitened circumstances owing to a heavy debt to the banker. Thanks to the great success of last year's festival dinner this debt had been cleared off, and 30 of the remaining beds had been reopened and were now in full swing. Owing to lack of funds they had had to refuse many urgent cases among the poor and needy of the East-end who had no other refuge to seek. The cost of the institution when all the beds were kept open was £12,000 per annum, while the assured income from the grant from the Prince of Wales's Hospital Committee, and also through the kindness of the various working men's associations—(applause)—was £4,000. It was for them to try to reduce that great shrinkage that night. From the Prince's fund they received in 1897 £900, and last year the committee of that fund determined to give them an annual subscription of £1,000. This certainly showed that the Committee of Inspection were satisfied with the management, and that they considered the work of the hospital was effectively carried out. (Applause.) The best thanks of all concerned were due to what had been done for the hospital by the working men's societies in the East-end. (Applause.) The Committee of Management had great pleasure in announcing that a ladies' committee had been formed for carrying out friendly work among the poor patients, whose hours would thus be brightened and their sufferings much relieved. The committee were most anxious to combat the fearful disease which the hospital existed to cope with, and were desirous of adopting the Continental open-air cure. They hoped to be able to rent a shelter in connexion with the hospital which would enable a certain number of patients to enjoy a modified form of the open-air treatment. This scheme, costing about £500, they hoped to be able to carry out by means of that night's collection. (Applause.) He called upon those present not only drink to, but also to aid in, the prosperity of the hospital. (Applause.)

The toast was honoured with much enthusiasm.

Mr. Horace G. Bowen, in proposing the "Health of the Chairman," referred particularly to the assistance rendered to charities by the members of the Rothschild family, remarking that the chairman belonged to a race and family they all respected, and he was sure it was the unanimous hope of all present that the position the house of Rothschild held in the City would be maintained for many years to come. The chairman himself took a deep interest in the welfare of this hospital, and deserved the warm thanks of all associated with it. (Applause.)

The Chairman briefly acknowledged the compliment, and the Ven. Archdeacon Sinclair then gave "The Treasurer and Committee," alluding to the serious nature of the disease which the hospital existed to combat, and to the need of proper provision for the treatment of cases which arose among those who could not afford to pay for medical aid. He spoke of the assistance generously rendered to the cause of charity by philanthropists in the City of London, and referred in warm terms to the kindly assistance which had been given to this hospital by the many societies of working men in the East-end. No matter what financial assistance was given, however, the success of the institution must depend upon the management being carried out on well-approved principles, and he was pleased to be able to speak very highly of the management of this hospital.

Sir Edward Sassoon, Bart., M.P., the treasurer, responded, and expressed a hope that the time would come when a tax would be levied upon betting for the benefit of charities, as was done on the other side of the Channel. Three millions sterling were said to change hands every year on the racecourses, and if a tax of 7½ per cent. were levied upon that money a quarter of a million would be provided annually for the relief of the sick and needy.

Mr. Joseph Benson, L.C.C., chairman of the committee, also replied.

"The Medical Staff," proposed by Mr. J. D. Farrell, was acknowledged by Dr. Heron.

While the dinner was being served some excellent music was contributed by the Leoni Ladies' Quintette.

During the evening the Secretary read the list of donations, the total of which reached £2,600, the chairman's contribution being 100 guineas.

